# Geometry-Based Synchrosqueezing S-Transform with Shifted Instantaneous Frequency Estimator Applied to Gearbox Fault Diagnosis

**DOI:** 10.3390/s25020540

**Published:** 2025-01-18

**Authors:** Xinping Zhu, Wuxi Shi, Zhongxing Huang, Liqing Shi

**Affiliations:** 1 School of Mechanical Engineering, Tiangong University, Tianjin 300387, China; zxp@heime-electric.com; 2 Guangzhou Metro Design and Research Institute Co., Ltd., Guangzhou 510010, China; huangzhongxing@dtsjy.com; 3 Heime (Tianjin) Electrical Engineering Systems Co., Ltd., Tianjin 301700, China; slq@heime-electric.com

**Keywords:** synchrosqueezing transform, instantaneous frequency, gearbox, S-transform, fault diagnosis

## Abstract

This paper introduces a novel geometry-based synchrosqueezing S-transform (GSSST) for advanced gearbox fault diagnosis, designed to enhance diagnostic precision in both planetary and parallel gearboxes. Traditional time-frequency analysis (TFA) methods, such as the Synchrosqueezing S-transform (SSST), often face challenges in accurately representing fault-related features when significant mode closely spaced components are present. The proposed GSSST method overcomes these limitations by implementing an intuitive geometric reassignment framework, which reassigns time-frequency (TF) coefficients to maximize energy concentration, thereby allowing fault components to be distinctly isolated even under challenging conditions. The GSSST algorithm calculates a new instantaneous frequency (IF) estimator that aligns closely with the ideal IF, thus concentrating TF coefficients more effectively than existing methods. Experimental validation, including tests on simulated signals and real-world gearbox fault data, demonstrates that GSSST achieves high robustness and diagnostic accuracy across various types of gearbox faults even in the presence of noise. Moreover, unlike conventional reassignment method, GSSST supports partial signal reconstruction, a key advantage for applications requiring accurate signal recovery. This research highlights GSSST as a promising and versatile tool for diagnosing complex mechanical faults and provides new insights for the future development of TFA methods in mechanical fault analysis.

## 1. Introduction

Gearboxes are critical components in various industries, serving essential functions such as torque transmission, speed variation, and direction change in machinery [1,2,3]. Their complex structures, composed of gears, shafts, bearings, and housings, are designed to ensure high productivity and longevity [4,5,6]. However, gearboxes are susceptible to various faults, such as wear, fatigue, misalignment, and pitting, which can lead to catastrophic failures if not detected in a timely manner [7,8]. Traditional fault diagnosis methods often struggle to effectively analyze nonstationary and nonlinear vibration signals generated by gearboxes, necessitating the development of advanced signal processing techniques.

Time-frequency analysis (TFA) methods have emerged as powerful tools for diagnosing mechanical faults, providing critical insights into the time-varying characteristics of signals [9,10,11,12]. Among these methods, short-time fourier transform (STFT), wavelet transform (WT), and S-transform (ST) have been extensively utilized for fault diagnosis in gearboxes. STFT, while beneficial, faces limitations in time-frequency resolution, especially for nonstationary signals. In contrast, WT offers multi-resolution analysis capabilities. ST combines the characteristics of both. However, they may still struggle to accurately depict transient features within complex signals such as close components or strongly time-varying features [13,14,15]. It is worth mentioning that the quadratic transform, such as Wigner–Ville distribution (WVD) [16] or Affine distribution [17], can produce more compact time-frequency representation (TFR) results. However, the cross terms in traditional quadratic TFA methods often limit their applicability in multi-component signal analysis.

In recent years, a series of post-processing techniques for TFA have been proposed. Most of these methods focus on manipulating the instantaneous frequency (IF) and group delay (GD) operators, achieving highly concentrated time-frequency representations (TFRs) by reassigning the energy in their vicinity. The reassignment method (RM) is an advanced TFA technique that aims to improve the concentration of energy in TFRs [18,19]. The reassignment method addresses this limitation of smeared representations in STFT or ST by relocating the energy from the surrounding area of a time-frequency point to its ideal location, effectively sharpening the TFR. In the reassignment process, two key operators are computed: the IF estimator and the GD estimator. These estimators provide a more accurate estimate of the energy’s true location in the time-frequency (TF) plane. The method reassigns the energy from a smeared region to the coordinates where the signal’s actual frequency and time characteristics are most concentrated, leading to a more precise representation of transient events and frequency changes. However, due to the loss of phase information, RM is unable to be reconstructed. SST, initially proposed in [20], can be seen as a refined version of the reassignment method only using IF estimator. SST has the advantage of being reversible, meaning that it allows for partial reconstruction of the original signal from TFRs, a feature that the traditional reassignment method does not provide [21,22].

Over the years, several extensions of SST have been proposed to further improve its concentration of TFR and adapt to increasingly complex signal analysis needs. The second-order synchrosqueezing transform (SST2) [23] extends the original SST by incorporating second-order phase information, providing even more accurate time-frequency localization. Building upon the principles of SST2, the high-order synchrosqueezing transform (HSST) [24] further generalizes the method by utilizing higher-order phase derivatives. This technique allows for even finer resolution in the TFR. In addition, demodulated synchrosqueezing transform (DSST) [25] introduces a pre-processing technique, termed demodulation transform, to better retrieval IF ridges. Instead of simply redistributing energy along these ridges, synchroextracting transform (SET) [26] directly isolates the ridges, making it an effective tool for the precise extraction of fault-related components in mechanical environments. In order to better showcase the geometric perspective of the post-processing techniques above, [27] provided a new method combining the advantages of SST and RM, which can achieve much more concentrated TFR and has the ability of reconstruction.

Inspired by the method proposed in [27], in this paper, a new algorithm named geometry-based synchrosqueezing S-transform (GSSST) is proposed for characterizing the gearbox signal with time-varying features. The proposed method starts from linear frequency-modulated signal, which simulates the trend of the IF trajectory by calculating the IF estimator and GD estimator at different TF points. Then, the new IF estimator can be calculated according to the obtained geometrical relationship of the IF trajectory. The new IF estimator more accurately approximates the ideal IF, enabling the TF coefficients to be reassigned with greater focus during the synchrosqueezing process. Additionally, the incorporation of a shifted instantaneous frequency estimator into the geometric reassignment process enhances the energy concentration of TFR while preserving signal reconstruction capability, which is essential for diagnostic precision and interpretability. The key contributions of this work are summarized as follows:

1. A novel extension of the geometric reassignment framework to ST, enabling the analysis of signals with varying TF characteristics while benefiting from ST’s inherent multi-resolution properties.
2. The introduction of a shifted IF estimator that improves the alignment of reassigned coefficients with the ideal IF trajectory, achieving a higher energy concentration in TFRs.
3. A comprehensive validation of the proposed GSSST method through simulations and experiments on real-world gearbox fault data, demonstrating its effectiveness in diagnosing complex mechanical faults under challenging conditions.

The remaining parts of this paper are organized as follows. In Section 2, the theoretical background of ST-based RM and synchrosqueezing S-transform (SSST) is recalled. The theoretical analysis and algorithm implementation are provided in Section 3. The comparative analysis of simulation examples and real examples of gearbox signal is listed in Section 4 and Section 5, respectively. Finally, Section 6 draws the conclusions.

## 2. Theoretical Background

### 2.1. S-Transform (ST)

For a given zero-mean signal *s*, the ST, with a frequency-dependent Gaussian window σ, can be expressed as:

(1) $$S\left(a,b\right)=\left|b\right|\int_{R}s\left(t\right)g\left(b\left(a-t\right),\sigma \right)e^{-ibt}dt$$

where the function of the Gaussian window in this paper is defined as $g\left(t,\sigma \right)\;=\;e^{-\frac{t^{2}}{2\sigma^{2}}}/\sqrt{2\pi }\sigma$, where σ is a time-spread parameter, adjusting the width of window, for ensuring the multi-resolution property of ST. The multi-component amplitude-modulated (AM) and frequency-modulated (FM) signal can be defined as:

(2) $$s\left(t\right)=A\left(t\right)e^{i\phi (t)},$$

where A(t) and ϕ(t) denote the amplitude and phase function the analytical signal *s*, respectively. For a weak time-varying signal whose first-order terms $O(A^{\prime }\left(t\right))$ and second-order terms $O(\phi^{\prime \prime }\left(t\right))$ are neglected, the analytical signal can be rewritten as:

(3) $$s\left(a\right)=A\left(a\right)e^{i(\phi \left(a\right)+\phi^{\prime }\left(a\right)\left(a-t\right))},$$

where $\exists \varepsilon ,\max\{|A^{\prime }\left(a\right)|,|\phi^{\prime \prime }\left(a\right)|\}\leq \varepsilon$ for ∀a. With Equation (3), the formula of ST can be deduced as:

(4) $$\begin{array}{cc} S(a,b) & =\left|b\right|\int_{R}A\left(a\right)e^{i(\phi \left(a\right)+\phi^{\prime }\left(a\right)\left(t-a\right))}g^{*}\left(b\left(t-a\right)\right)\cdot e^{-ib(t-a)}dt\\ \; & =|b|A\left(a\right)e^{i\phi (a)}\int_{R}g^{*}\left(b\left(t-a\right)\right)\cdot e^{-i(b-\phi^{\prime }\left(a\right))\left(t-a\right)}d\left(t-a\right)\\ \; & =|b|A\left(a\right)e^{i\phi (a)}{\hat{g}}^{*}\left(b\left(b-\phi^{\prime }\left(a\right)\right)\right). \end{array}$$

Due to $\hat{g}$ being the zero-centered compact function, the coefficients of ST result smears in the TF region $b\in [\phi^{\prime }\left(a\right)-\Delta ,\phi^{\prime }\left(a\right)+\Delta ]$. Therefore, reassigning the smeared coefficients for achieving high concentrated TFR result is important, which is also the motivation of RM.

### 2.2. ST-Based RM

As a post-processing method of linear TF transform, RM aims to achieve high concentrated TFR by reassigning the coefficients from (a,b) to $\left(\hat{a}\left(a,b\right),\hat{b}\left(a,b\right)\right)$. For S-transform, the IF estimator and GD estimator are calculated as follows [28,29]:

(5) a^(a,b)=a−ReSag(a,b)S(a,b),

(6) b^(a,b)=b+ImSdg(a,b)S(a,b).

where $S^{ag}\left(a,b\right)$ and $S^{dg}\left(a,b\right)$ represent the ST results obtained using alternative Gaussian window functions ag and dg and ag and dg represent a×g(a) and the derivative of g(t), respectively. Considering that RM’s operation involves integrating the absolute value of TFR results, it cannot be reconstructed back to the original signal.

**Figure 1:**
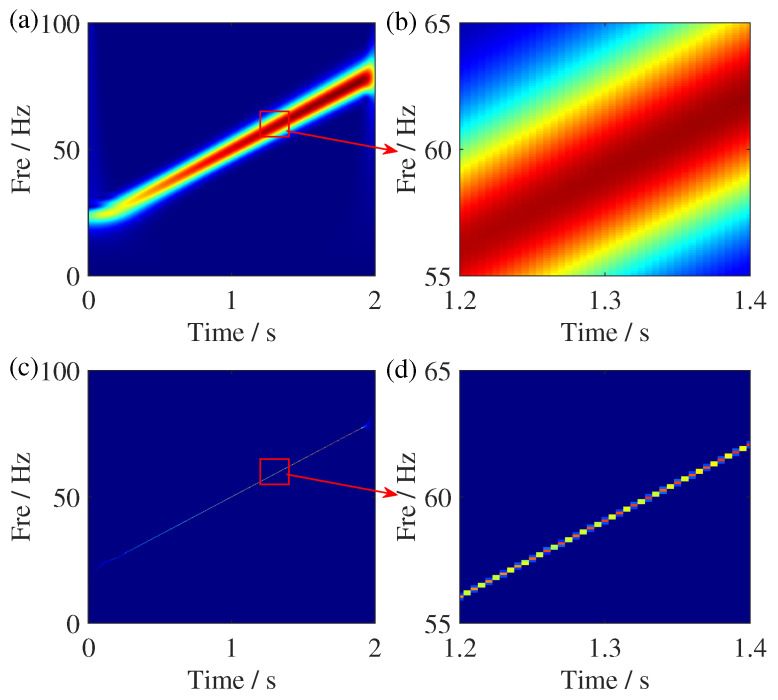
TFR comparisons of the FM signal. (**a**) ST result, (**b**) zoom of the ST result, (**c**) RM result, and (**d**) zoom of the RM result.

**Figure 2:**
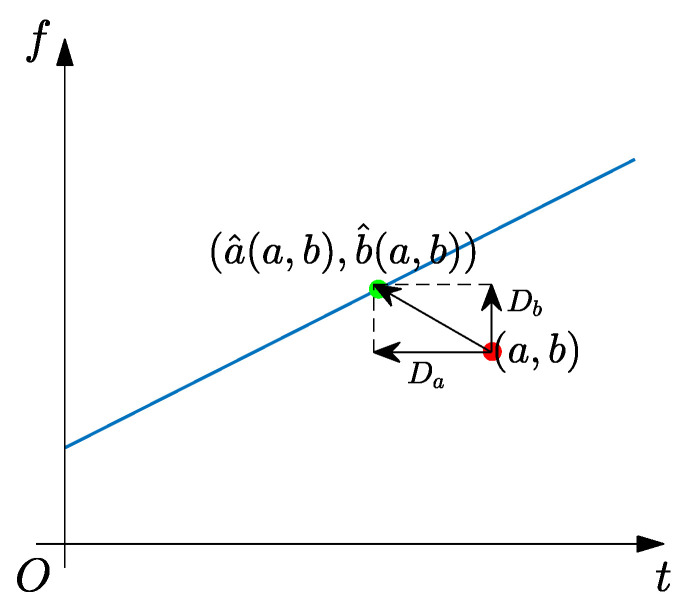
Geometrical relationship of RM’s principle. The arrow points the reassignment direction of TF coefficients.

In Figure 1, we provide a comparison of TFR results between ST and RM for a signal with linear FM mode, where Fre is the abbreviation of frequency. From the TFR result of ST, it can be seen that its energy smears around the ideal GD. The TFR result of RM shows a high energy concentration, indicating that the TF coefficients of its calculated result have been well reassigned to the ideal TF location. According to the reassignment manner of TF coefficients in the RM calculation process, the corresponding geometrical relationship of its principle is shown in Figure 2.

In Figure 2, we assume that (a,b) is a TF coefficient at an arbitrary position in the ST result and $\left(\hat{a}\left(a,b\right),\hat{b}\left(a,b\right)\right)$ is the corresponding point composed of IF and GD estimators. For time-varying signals, most TF positions fall on the IF trajectory. Therefore, RM can provide an ideal TFR result by reassigning TF coefficients along TF directions.

### 2.3. SSST

**Figure 3:**
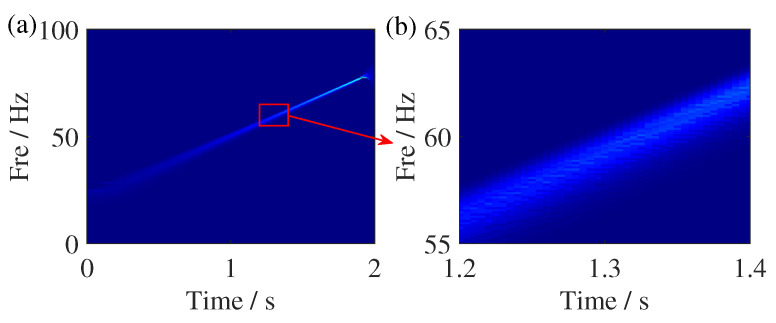
TFR comparisons of the FM signal. (**a**) SSST result and (**b**) zoom of the SSST result.

**Figure 4:**
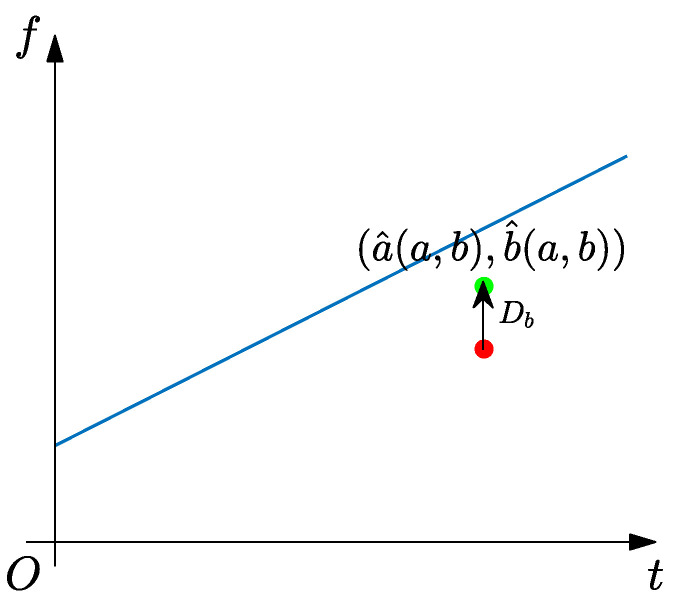
Geometrical relationship of SSST’s principle. The arrow points the reassignment direction of TF coefficients.

As a method developed from RM, SSST only reassigns TF coefficients in the frequency direction, i.e., $\left(a,b\right)\to (a,\hat{b}\left(a,b\right))$. Then, the formula of SSST and its reconstruction can be expressed as:

(7) $$SSST\left(a,\omega \right)=\int_{R}S\left(a,b\right)\delta \left(\omega -\hat{b}\left(a,b\right)\right)db,$$

(8) $$s\left(a\right)={\left(2\pi I_{g}\left(0\right)\right)}^{-1}\int_{R}SSST\left(a,\omega \right)d\omega ,$$

where $I_{g}\left(a\right)=\int_{R}\left|b\right|A\left(t\right){\hat{g}}^{*}\left(b\left(b-\phi^{\prime }\left(a\right)\right)\right)db$ and δ represents Dirac function. The TFR result of SSST is given in Figure 3. Although the energy concentration of SSST has improved compared to the TFR results of ST, there is still significant energy smeared, which is more severe at low frequencies. From Figure 4, it can be seen that the reason for this phenomenon is that a single IF estimator has a certain distance from the ideal IF, which results in some TF coefficients only being reassigned to the vicinity of IF.

## 3. GSSST

From the geometric relationship in Figure 4, it can be seen that SSST cannot provide satisfactory TFR results as compared to RM. However, the reassignment manner of SSST, only concentrating energy in time direction, maintains the signal reconstruction ability. Therefore, ensuring the reconstruction ability of TFA algorithm while accurately reassigning their TF coefficients to ideal IF is a topic worth exploring, which is also the motivation in this paper.

**Figure 5:**
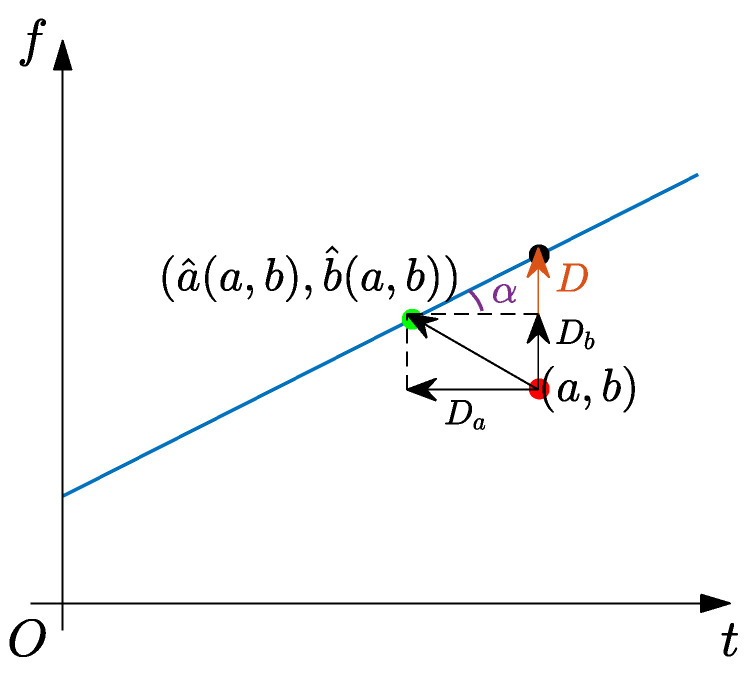
Geometrical relationship of GSSST’s principle. The arrow points the reassignment direction of TF coefficients.

Inspired by the geometrical relationship of the reassignment manner of TF coefficients, this paper integrates the advantages of RM and SSST. GSSST utilizes reassignment estimators of RM to redefine IF estimator, then performs reassignment in the manner of SSST with new IF estimator. The principle of GSSST is shown in Figure 5 and its exact procedure can be explained as follows: (1) calculating the horizontal inclination angle of ideal IF through two close reassignment estimators of RM and (2) defining new IF estimator based on calculated angle and $D_{a}$. According to Figure 5, the tangent of horizontal inclination angle of ideal IF can be calculated as:

(9) $$\tan\alpha =\frac{\hat{b}\left(a+\Delta ,b\right)-\hat{b}\left(a,b\right)}{\hat{a}\left(a+\Delta ,b\right)-\hat{a}\left(a,b\right)},\hat{a}\left(a+\Delta ,b\right)-\hat{a}\left(a,b\right)\neq 0.$$

Equation (9) calculates the inclination angle tanα of the ideal IF trajectory by using two adjacent reassignment points $\left(\hat{a}\left(a,b\right),\hat{b}\left(a,b\right)\right)$ and $\left(\hat{a}\left(a+\Delta ,b\right),\hat{b}\left(a+\Delta ,b\right)\right)$. The inclination angle reflects the rate of frequency variation over the temporal offset Δ, effectively capturing the local geometric trend of the IF trajectory. This geometric relationship serves as the foundation for refining the IF estimator by minimizing the lateral deviation between the current reassignment point and the ideal trajectory. Therefore, the lateral distance between the original IF and ideal IF estimator can be determined based on the geometrical relationship. Then, we can get a new IF estimator that is almost equivalent to the ideal IF. Their formulas can be expressed as:

(10) $$D=D_{x}\tan\alpha =\left(a-\hat{a}\left(a,b\right)\right)\tan\alpha ,$$

(11) $$b_{g}\left(a,b\right)=\hat{b}\left(a,b\right)+D.$$

Based on the inclination angle tanα, the lateral deviation *D* between the original IF and the ideal IF trajectory is determined using Equation (10). This deviation quantifies the misalignment of the reassigned TF coefficient relative to the ideal trajectory. By incorporating *D* into the IF estimation (Equation (11)), the refined IF estimator $b_{g}\left(a,b\right)$ effectively aligns the reassigned coefficients with the ideal trajectory, thereby enhancing the energy concentration in the TFR. This refinement is particularly beneficial for signals with rapidly varying frequency components, as it provides a more accurate localization of TF energy. According to the new IF estimator, the formula of GSSST can be written as:

(12) $$GSSST\left(a,\omega \right)=\int_{R}S\left(a,b\right)\delta \left(\omega -{\hat{b}}_{g}\left(a,b\right)\right)db.$$

**Figure 6:**
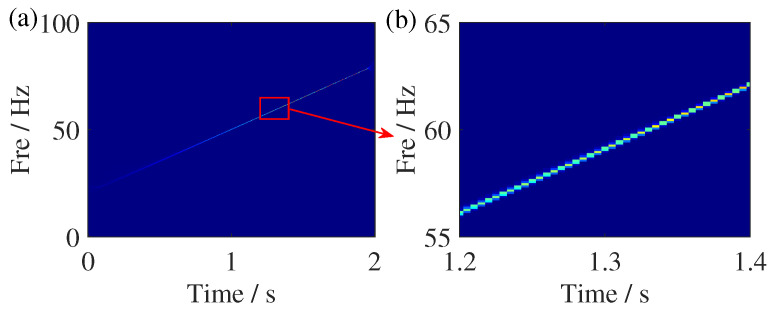
TFR comparisons of the FM signal. (**a**) GSSST result and (**b**) zoom of the GSSST result.

The TFR result of GSSST of the above signal is given in Figure 6. It can be seen that the energy concentration of GSSST is significantly improved compared to SSST. The corresponding Renyi entropies of above TFR results are listed in Table 1. Apparently, the Renyi entropy of GSSST is fairly close to the second to RM, which also indicates that the proposed method achieves a highly concentrated TFR result.

In addition, the parameter Δ plays a crucial role in defining the geometric relationship between local TF points and the ideal IF trajectory within the synchrosqueezing process. Specifically, Δ represents a small temporal offset used to compute the inclination angle tanα of the ideal IF trajectory. This parameter serves to capture the local variations of the IF trajectory by quantifying the horizontal slope between two reassignment points in the TF plane. The choice of Δ significantly affects the performance of proposed method. A larger Δ may result in an inaccurate geometric representation, especially for nonstationary signals with rapid variations, while a smaller Δ improves the accuracy of the geometric relationship but may amplify noise sensitivity. Therefore, Δ must be carefully tuned to balance the trade-off between robustness and precision.

Moreover, the biggest advantage of the proposed method over RM is that it allows the signal to be reconstructed, which can be expressed as:

(13) $$s\left(a\right)={\left(2\pi I_{g}\left(0\right)\right)}^{-1}\int_{R}GSSST\left(a,\omega \right)d\omega .$$

## 4. Simulated Validation

The simulation validation of mono-component has been attached to the TFA of various methods in Section 2 and Section 3. In this section, for validating the performance of proposed method for planetary gearboxes, the signal model in [30,31] is constructed. Without loss of generality, we neglect the initial phases and carrier frequency, only focusing on the fundamental frequency of modulating components. The signal model with sun gear fault in planetary gearboxes is expressed as:

(14) $$\begin{array}{cc} x(t) & =\left\{\underset{\mathrm{AM}\;\mathrm{by}\;\mathrm{sun}\;\mathrm{rotation}}{\underset{︸}{1-\cos\left[2\pi \int f_{sr}\left(t\right)dt\right]}}\right\}\cdot \left\{\underset{\mathrm{AM}\;\mathrm{by}\;\mathrm{sun}\;\mathrm{fault}}{\underset{︸}{1+A\cos\left[2\pi \int f_{s}\left(t\right)dt+\phi \right]}}\right\}\\ & \cdot \cos\left\{2\pi \int f_{m}\left(t\right)dt+\underset{\mathrm{FM}\;\mathrm{by}\;\mathrm{sun}\;\mathrm{fault}}{\underset{︸}{B\sin\left[2\pi \int f_{s}\left(t\right)dt+\theta \right]}}\right\}, \end{array}$$

where amplitude-modulation magnitude A=1.4, frequency-modulation magnitude B=0, and initial phrases ϕ=θ=0. In order to simulate the linear changes of modes and adapt to the scenarios that the proposed method is suitable for, we let the rotating frequency of sun gear $f_{s}r\left(t\right)$, sampled at a frequency of 2000 Hz, linearly increased from 35 to 50 Hz, then decreased to 35 Hz over 1 s. The sun gear fault characteristic frequency and gear meshing frequency are assumed as $f_{s}\left(t\right)=\left(10/3\right)f_{s}r\left(t\right)$ and $f_{m}\left(t\right)=\left(40/3\right)f_{s}r\left(t\right)$, respectively. According to the planetary gearboxes fault signal given in Equation (14), there are nine main components, i.e., $\left[f_{m},f_{m}\pm f_{sr},f_{m}+f_{s}-f_{sr},f_{m}-f_{s}+f_{sr},f_{m}\pm f_{s},f_{m}+f_{s}+f_{sr},f_{m}-f_{s}-f_{sr}\right]$, in TFR ideally.

**Figure 7:**
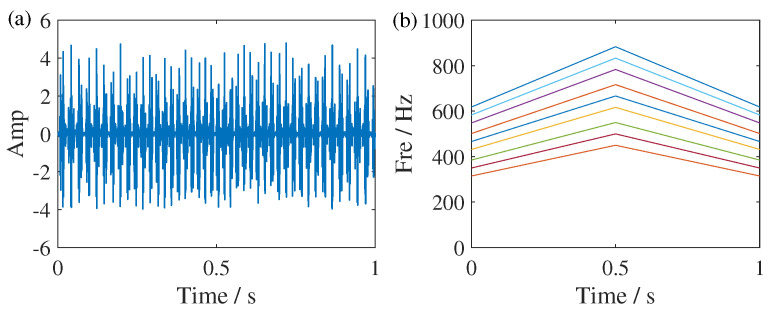
Time domain and frequency domain representations of the simulated signal. (**a**) The time-domain waveform and (**b**) ideal IF trajectories of the simulated signal. The different color represents the IF trajectories of different components of the signal in (**a**).

**Figure 8:**
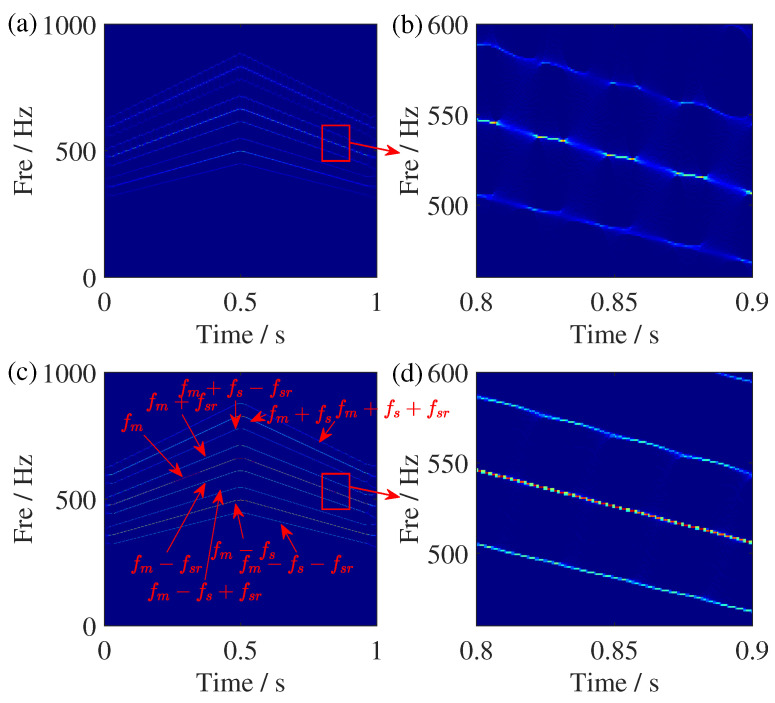
TFR comparisons of the simulated signal. (**a**) SSST result, (**b**) zoomed view of the SSST results, (**c**) GSSST result, and (**d**) zoomed view of the GSSST result. The red arrow in (**c**) points to IF trajectories with processed signal. Others is the zoomed view in red box.

**Figure 9:**
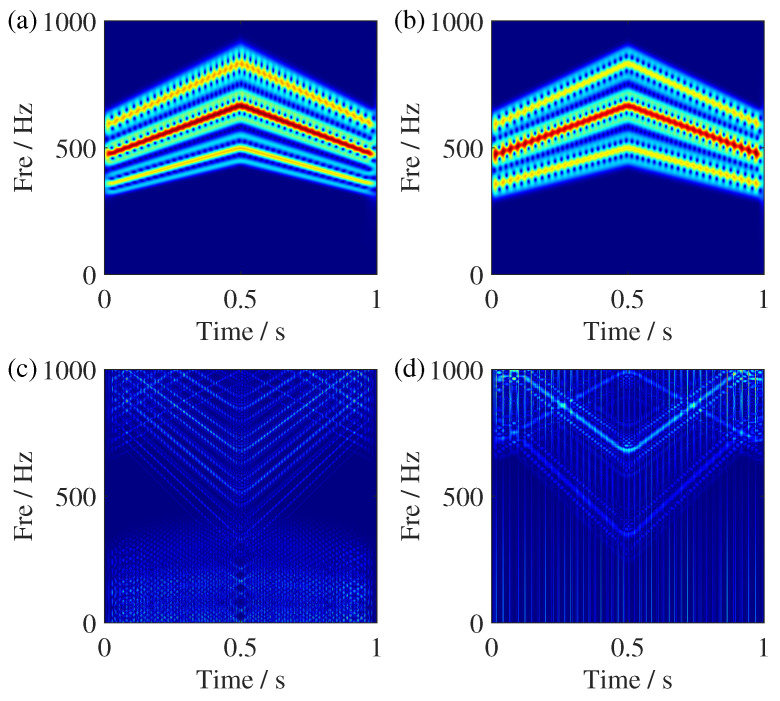
TFR comparisons of the simulated signal. (**a**) ST result, (**b**) STFT, (**c**) WVD result, and (**d**) RIDT result. The different color is the region where the corresponding value of TF coefficients.

Figure 7 shows the time-domain waveform and ideal IF trajectories simulated signal, where Amp is the abbreviation of amplitude. The TFR results of SSST and GSSST are given in Figure 8. It can be seen that both of them can accurately characterize the change law of each component. In the zoom view of SSST result, there still exists slight energy smearing when the IFs become closer. In contrast, the GSSST result achieves high energy concentration across the entire speed range. The mode of $f_{m},f_{m}\pm f_{sr}$ in Figure 8c is the normal state of the planetary gear vibration signal. The others are the fault frequencies caused by the sun gear fault. For the sake of more comparisons, the TFR results calculated by some representative TFA methods, such as ST, STFT, WVD, and RIDT (reduced-interference distribution with triangular kernel), are given in Figure 9. It can be seen that ST result exhibit significant energy smearing without synchrosqueezing transform, particularly in the close IF region. The STFT result shows more serious fuzzy energy. Moreover, the energies of TFR results given by quadratic transforms, i.e., WVD and RIDT, are more compact, but its own cross terms will influence the expression of complex signals. In other view, the Renyi entropies of the above TFRs are given in Table 2, too. The GSSST get the lowest Renyi entropy, which means the proposed method achieves the most concentrated TFR from quantitative analysis.

**Figure 10:**
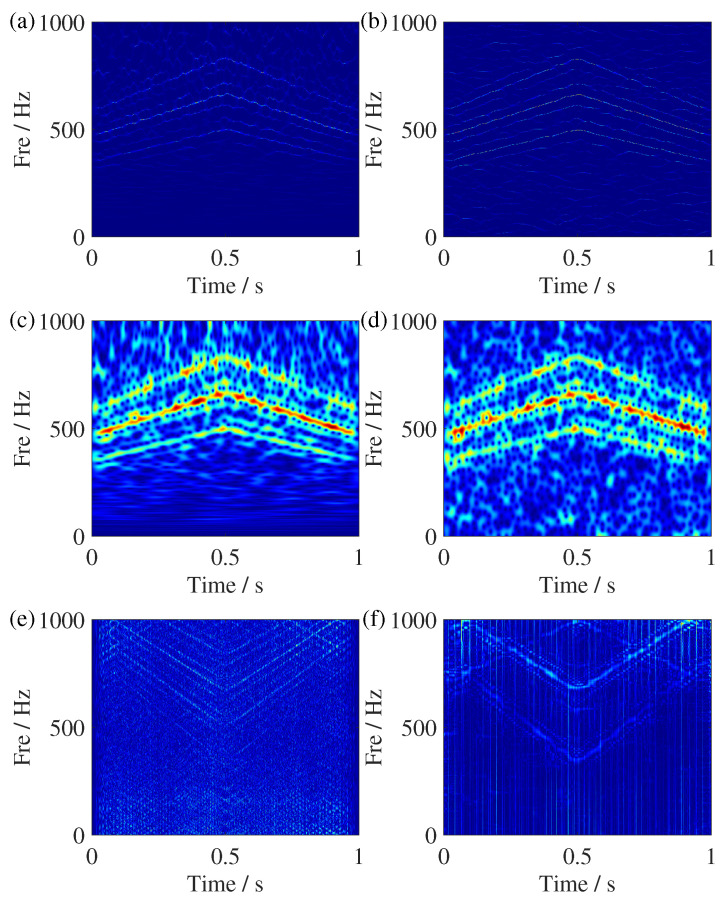
TFR comparisons of the simulated signal with noise. (**a**) SSST result, (**b**) GSSST result, (**c**) ST result, (**d**) STFT result, (**e**) WVD result, and (**f**) RIDT result.

**Figure 11:**
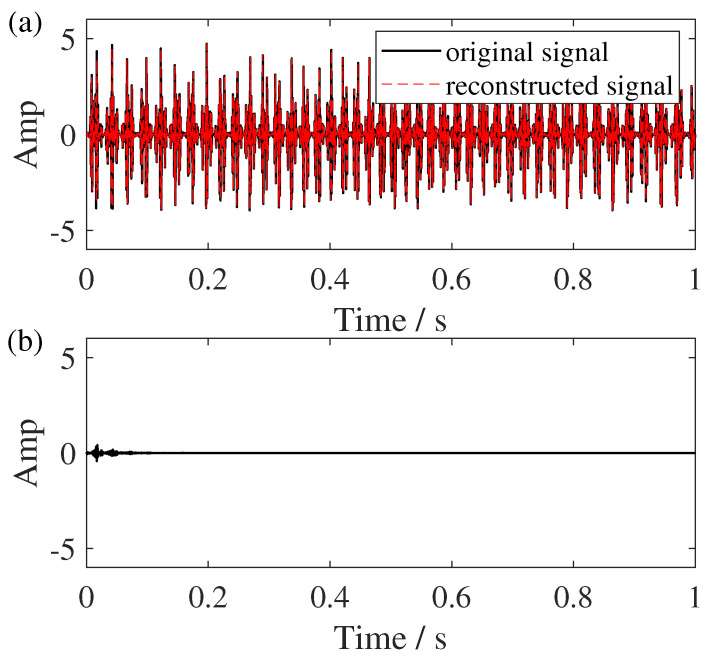
Reconstruction analysis. (**a**) The reconstruction result of GSSST and (**b**) its reconstruction error.

In addition, we added 2 dB white noise in the constructed sun gear fault signal to discuss the noise adaptability of the proposed method. The TFR results are shown in Figure 10. Obviously, the results of GSSST retain clear representations of each component, and other TFRs exhibit varying degrees of energy smearing. In the end, the reconstruction of the pure signal is given in Figure 11. The error between the original signal and result proves GSSST can achieve rough reconstruction. Only a small difference can be seen in the first two oscillation cycles.

## 5. Experimental Validation

In this section, we focus on the application of SSST for fault feature extraction in planetary gearboxes and parallel gearboxes, which are often used together. The wind turbine planetary gearbox fault data from BJTU [32] and the two-stage parallel gearbox fault data from MCC5-THU [33] are used to further validate the effectiveness of the proposed method. It is important to highlight that no other factors were identified that could replicate the results observed under specific fault conditions in the two sets of experimental data discussed in this section.

### 5.1. Application for Planetary Gearbox Fault Data

**Figure 12:**
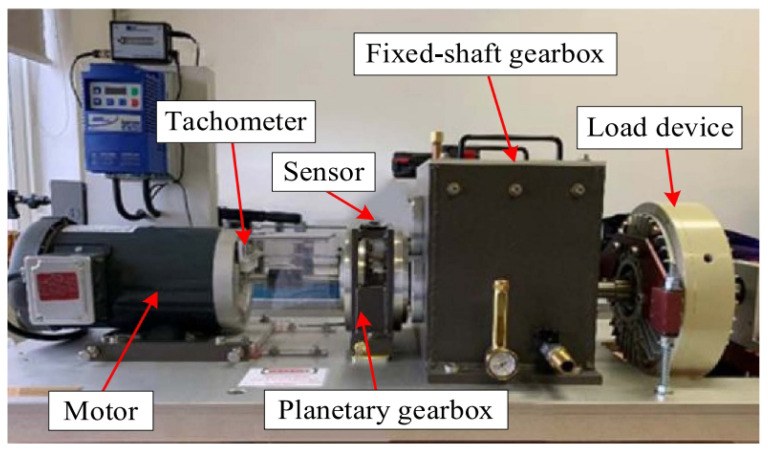
The experimental setup of the wind turbine planetary gearbox.

The experimental setup, shown in Figure 12, includes a driving motor, tachometer, planetary gearbox, fixed-shaft gearbox, and load device. Vibration data are captured using accelerometers mounted on the planetary gearbox, with signals from the X axis and Y axis being used for analysis, and the data are sampled at 48 kHz. In the planetary gearbox, four planetary gears rotate around a sun gear. The experiment involves four planetary gear faults (e.g., wear, missing teeth, cracks, and broken teeth) with eight speed conditions. All these gear faults can be recognized by proposed method. For the purpose of this study, we focus on the X-axis vibration signals running at 55 Hz, corresponding to the broken teeth fault in the planetary gearbox.

**Figure 13:**
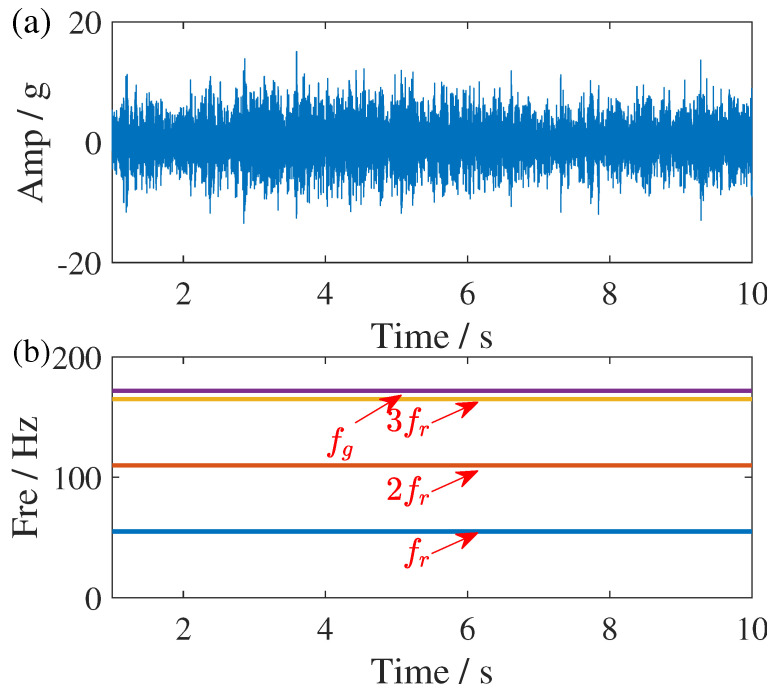
Time domain and its primary components representations of the gearbox fault signal. (**a**) Waveform of the vibration signal and (**b**) its primary components. The different color represents the IF trajectories of different components of the signal in (**a**).

The structure parameters of this planetary gearbox are listed in Table 3, where $f_{r}$ is denoted as sun gear rotating frequency. Based on the information in Table 3, it can be observed that the fault frequency of the sun gear is approximately 171.875 Hz when the rotational frequency is 55 Hz. Based on this observation, the time-domain waveform of the 1–10 s of the signal and the IF of the primary components in the low-frequency range are illustrated in Figure 13. In this figure, unit *g* indicate the strength of acceleration, $f_{g}$ represents the fault frequency, while the other three components correspond to the rotational frequency and its harmonics.

**Figure 14:**
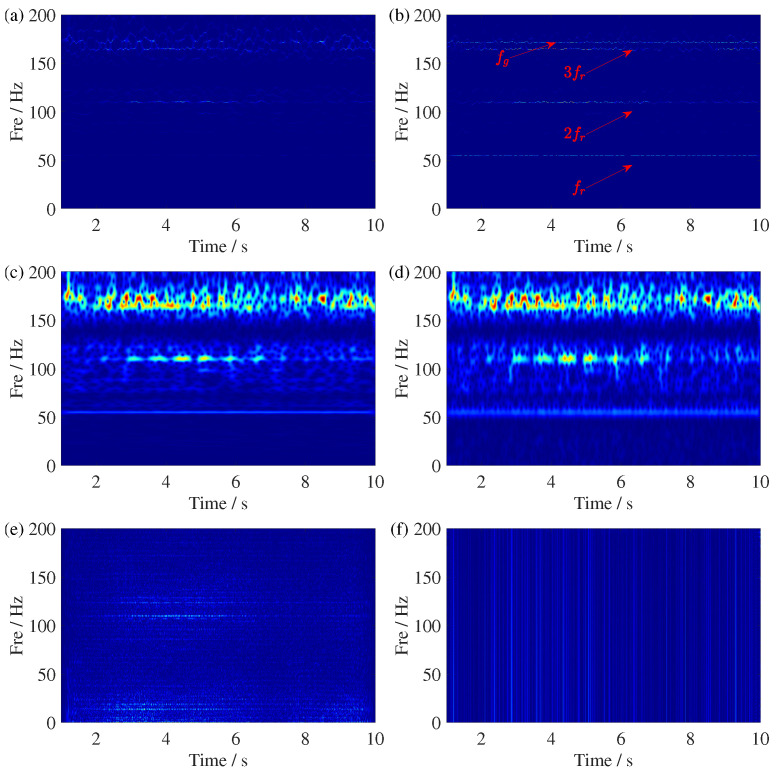
TFR comparisons of the gearbox fault signal. (**a**) SSST result, (**b**) GSSST result, (**c**) ST result, (**d**) STFT result, (**e**) WVD result, and (**f**) RIDT result. The red arrow points to IF trajectories with processed signal.

The TFR results of the gearbox fault signal using GSSST and several comparison methods are shown in Figure 14. The most significant comparison is between SSST in Figure 14a and GSSST in Figure 14b, as GSSST is a direct improvement based on SSST. Both TFR results clearly display the $f_{r}$ and $2f_{r}$ modes. However, GSSST shows a higher concentration of energy. More importantly, the focus is on how well the TFA extracts the fault mode, specifically the mode at $f_{m}=171.785$ Hz. Thanks to the higher energy concentration and better mode preservation, GSSST can distinguish the $2f_{r}$ and $f_{m}$ modes with clarity, with only minor mode overlap at certain time points. In contrast, SSST completely fails to capture the overlap between $3f_{4}$ and $f_{m}$. Moreover, the TFR results of STFT and ST also only extract the $f_{r}$ and $2f_{r}$ components, but with more severe energy dispersion. Due to their inherent limitations and lack of advanced energy concentration techniques, WVD and RIDT struggle to extract the fault mode, making them less suitable for planetary gearbox fault diagnosis.

**Figure 15:**
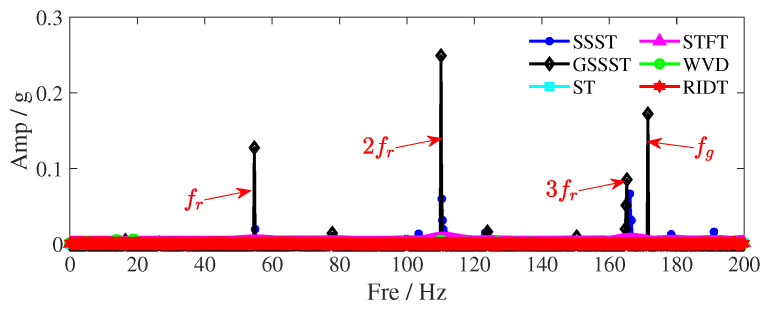
TF slices of TFR results at 4 s. The red arrow points to the Amp of TF coefficients at 4 s.

To quantitatively compare the energy concentration repeatedly mentioned, Table 4 presents the Renyi entropy values for the TFR results in Figure 14. The significantly lower Renyi entropy of GSSST compared to other methods demonstrates the improvement in energy concentration achieved by the proposed method over SSST. Additionally, Figure 15 plots the TF slices across the entire frequency range at the 4 s mark for each TFR result, providing a clear depiction of the energy dispersion among the components. It is evident that GSSST exhibits narrower value distributions and higher peak energy for each component, further confirming its superior energy concentration.

### 5.2. Application for Parallel Gearbox Fault Data

In some applications, parallel gearboxes and planetary gearboxes can be used in combination. For example, in some high load, high torque applications, planetary gearboxes can be used as primary reducers, while parallel axis gearboxes are used for subsequent deceleration or torque transmission. This combination can effectively utilize the advantages of two types of gearboxes to achieve efficient and reliable power transmission. As a result, a parallel gearbox fault signal under the speed-varying condition is used in this subsection.

**Figure 16:**
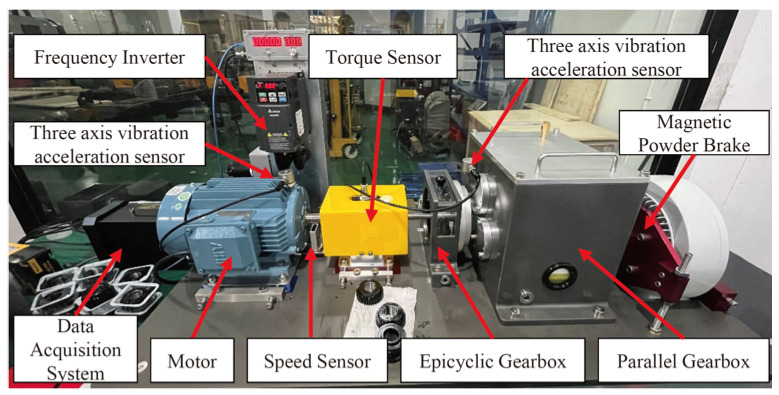
The gearbox test rig.

**Figure 17:**
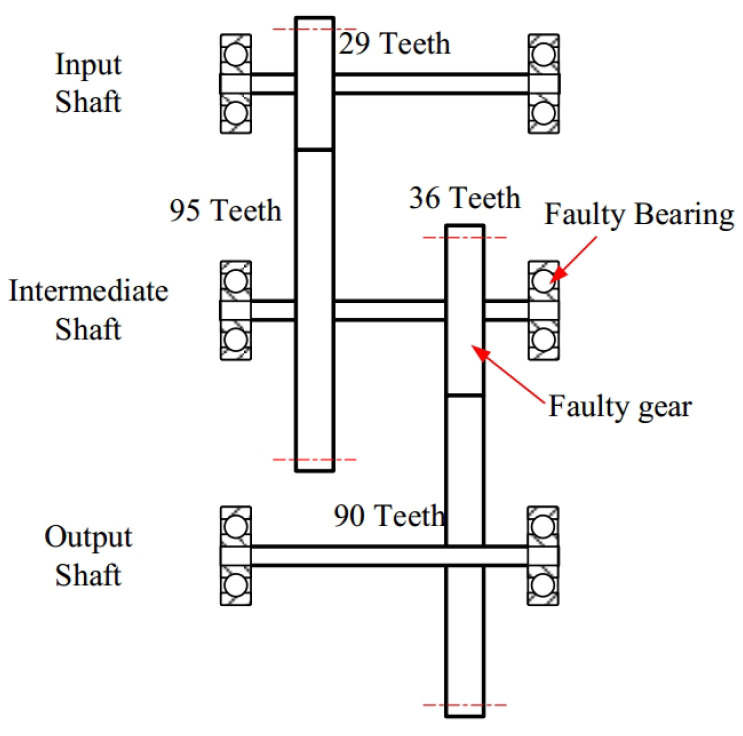
The structure diagram of the parallel gearbox.

**Figure 18:**
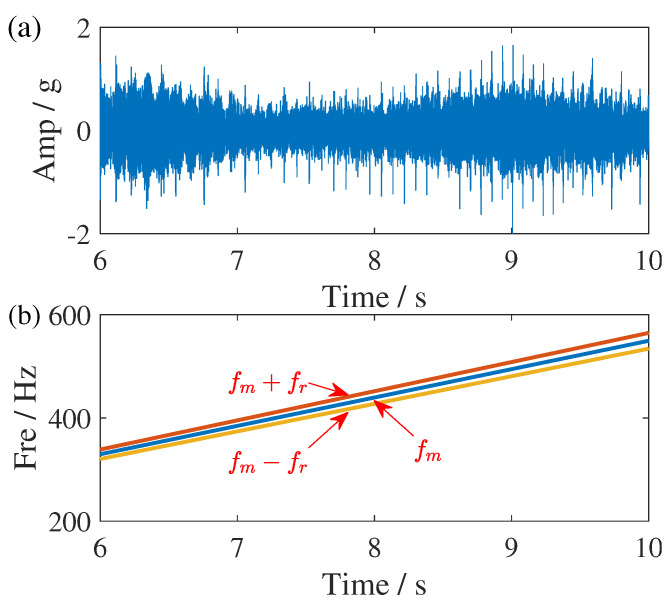
Time domain and IF representations of the gearbox fault signal. (**a**) The time waveform of vibration signal at z-axes and (**b**) the ideal mesh frequency and corresponding IF. The red arrow points to ideal IF trajectories with processed signal.

The experimental platform, depicted in Figure 16, consists of a 2.2 kW three-phase motor, a torque sensor, a two-stage parallel gearbox, a magnetic powder brake for applying load, and a control system. This setup simulates various fault conditions of the 36-tooth gear on the intermediate shaft and its adjacent support bearings. Vibration data along the x-, y-, and z-axes are captured by two three-axis accelerometers placed on the motor and gearbox. The signals are sampled at 12.8 kHz. Similarly, we only select the speed-varying condition with miss teeth fault but without bearing fault. The time waveform of z-axes acceleration signal in 6–10 s and its frequency spectrum are given in Figure 17. The rotational frequency of input shaft in this time slot is linear increasing from 30 Hz to 50 Hz. According to the structure diagram of the gearbox, given in Figure 17, the meshing frequency $f_{m}$ of the small gear on the intermediate shaft will increase from 329.7 Hz to 549.5 Hz correspondingly. The sidebands caused by the fault will be distributed on both sides of the meshing frequency based on the rotational frequency difference of the intermediate shaft. The corresponding time waveform and its ideal one-order feature frequency are shown in Figure 18.

**Figure 19:**
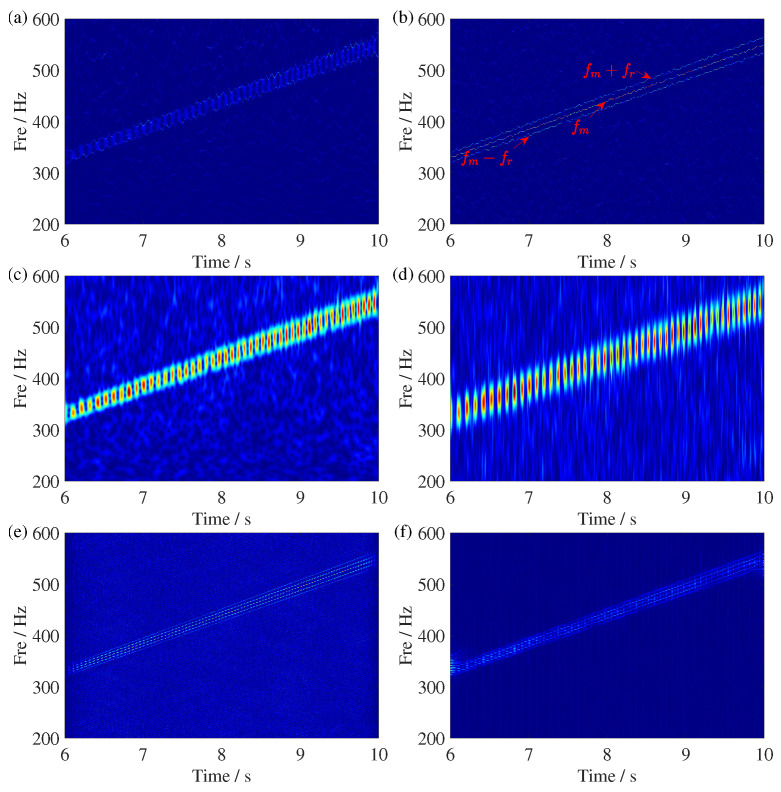
TFR comparisons of the gearbox fault signal. (**a**) SSST result, (**b**) GSSST result, (**c**) ST result, (**d**) STFT result, (**e**) WVD result, and (**f**) RIDT result. The red arrow points to IF trajectories with processed signal.

Similarly, we present the results of applying GSSST and other TFA methods to the gearbox fault signal in Figure 19. For ST and STFT, only the blurred meshing frequency can be observed, with sidebands completely overlapping the meshing frequency. SSST shows partial mode overlap, while some components near the meshing frequency can be identified, it fails to extract the precise characteristic frequencies. Fortunately, the proposed GSSST not only clearly separates the meshing frequency from the upper and lower sidebands, but its impressive energy concentration also enables accurate extraction of sideband frequency variations. In contrast, WVD and RIDT results exhibit significant cross-terms. In conclusion, the proposed GSSST proves effective not only for fault diagnosis in planetary gearboxes but also as a reliable tool for diagnosing faults in parallel gearboxes.

## 6. Conclusions

In this study, we introduced the GSSST as an innovative method for analyzing nonstationary signals, particularly for applications in gearbox fault diagnosis. Compared with conventional SSST, GSSST achieves superior energy concentration and enables precise extraction of fault characteristics, effectively distinguishing between closely spaced frequencies, such as meshing frequencies and sidebands, which are commonly encountered in gearbox signals. By employing a novel geometric reassignment framework, GSSST redefines the IF estimator to improve the accuracy of TF coefficient reassignment, resulting in a clearer and better TFR.

Validated on both simulated data and real-world fault signals, GSSST proves effective in identifying multiple fault types, including miss teeth and broken teeth, under varying operational conditions and noise levels. Additionally, unlike traditional reassignment techniques that cannot reconstruct signals, GSSST’s signal reconstruction capability addresses this limitation, facilitating accurate signal recovery when needed. The results indicate that GSSST provides a powerful and adaptable approach for diagnosing complex mechanical faults, showcasing its potential for broader applications in mechanical diagnostics. These findings support GSSST as a significant advancement in TFA and as a foundation for future research into increasingly complex fault diagnosis requirements across diverse industrial settings. In summary, the practical implications of this methodology extend to a wide range of nonstationary signal analysis tasks, particularly in the fields of mechanical fault diagnosis, condition monitoring, and structural health assessment. The proposed method offers a promising tool for addressing complex challenges in TFA and lays the groundwork for future research into more adaptable and efficient diagnostic algorithms for real-world applications. Future work will explore the integration of the proposed method with state-of-the-art techniques to tackle increasingly complex diagnostic requirements.

## Figures and Tables

**Table 1:** Renyi entropies by the above TFA methods.

**TFA method**	ST	RM	SSST	GSSST
**Renyi entropy**	16.5207	10.4725	13.8311	10.7459

**Table 2:** Renyi entropies by the above TFA methods.

**TFA method**	ST	SSST	GSSST	STFT	WVD	RIDT
**Renyi entropy**	15.8438	12.1053	10.1574	19.2477	19.1409	18.9354

**Table 3:** Structure parameters of a planetary gearbox.

Sun Gear Tooth Number	Ring Gear Tooth Number	Planetary Gear Tooth Number	Planetary Gear Numberr	Fault Frequency of Sun Gear
28	100	36	4	(25/8)$f_{r}$

**Table 4:** Renyi entropies by the above TFA methods.

**TFA method**	ST	SSST	GSSST	STFT	WVD	RIDT
**Renyi entropy**	18.1538	13.2065	11.0169	21.0169	21.0670	20.9345

## Data Availability

Dataset available on request from the authors.

## References

[1] Saufi S.R., Ahmad Z.A.B., Leong M.S., Lim M.H. (2020). Gearbox fault diagnosis using a deep learning model with limited data sample. IEEE Trans. Ind. Inform..

[2] García P.L., Crispel S., Saerens E., Verstraten T., Lefeber D. (2020). Compact gearboxes for modern robotics: A review. Front. Robot. AI.

[3] El Yousfi B., Soualhi A., Medjaher K., Guillet F. (2022). Electromechanical modeling of a motor–gearbox system for local gear tooth faults detection. Mech. Syst. Signal Process..

[4] Liu B., Riemenschneider S., Xu Y. (2006). Gearbox fault diagnosis using empirical mode decomposition and Hilbert spectrum. Mech. Syst. Signal Process..

[5] Kumar A., Gandhi C.P., Zhou Y., Kumar R., Xiang J. (2020). Latest developments in gear defect diagnosis and prognosis: A review. Measurement.

[6] Xu M., Han Y., Sun X., Shao Y., Gu F., Ball A.D. (2022). Vibration characteristics and condition monitoring of internal radial clearance within a ball bearing in a gear-shaft-bearing system. Mech. Syst. Signal Process..

[7] Gu H., Liu W.Y., Gao Q.W., Zhang Y. (2021). A review on wind turbines gearbox fault diagnosis methods. J. Vibroeng..

[8] Wu H., Zhi S., Fang Q., Liu Y., Wang T., Cheng W., Chu F. (2024). Synchronous Decomposition Match-Reassigning Transform and Its Application in Planetary Gearbox Fault Diagnosis. Meas. Sci. Technol..

[9] Lei Y., Lin J., Zuo M.J., He Z. (2014). Condition monitoring and fault diagnosis of planetary gearboxes: A review. Measurement.

[10] Vitor A.L.O., Goedtel A., Castoldi M.F., Souza W.A., Bazan G.H. (2023). Induction machine fault diagnosis with quadratic time–frequency distributions: State of the art. IEEE Trans. Instrum. Meas..

[11] Liu F., Shang Z., Gao M., Li W., Pan C. (2023). Bearing failure diagnosis at time-varying speed based on adaptive clustered fractional Gabor transform. Meas. Sci. Technol..

[12] Qin R., Huang J., Zhang Z., Du Z., Xiang X., Yu Y., Wen G., He W., Chen X. (2024). An adaptive cepstrum feature representation method with variable frame length and variable filter banks for acoustic emission signals. Mech. Syst. Signal Process..

[13] Li H., Wu X., Liu T., Li S. (2023). Rotating machinery fault diagnosis based on typical resonance demodulation methods: A review. IEEE Sens. J..

[14] Liu W., Liu Y., Li S. (2024). Time-reassigned multisynchrosqueezing of the S-transform for seismic time-frequency analysis. Acta Geophys..

[15] Liu W., Liu Y., Li S. (2022). Demodulated multisynchrosqueezing S transform for fault diagnosis of rotating machinery. IEEE Sens. J..

[16] Zhou Y., Chen J., Dong G.M., Xiao W.B., Wang Z.Y. (2011). Wigner–Ville distribution based on cyclic spectral density and the application in rolling element bearings diagnosis. Proc. Inst. Mech. Eng. Part C J. Mech. Eng. Sci..

[17] Feng Z., Liang M., Chu F. (2013). Recent advances in time–frequency analysis methods for machinery fault diagnosis: A review with application examples. Mech. Syst. Signal Process..

[18] Kodera K., Gendrin R., Villedary C. (1978). Analysis of time-varying signals with small BT values. IEEE Trans. Acoust. Speech Signal Process..

[19] Auger F., Flandrin P. (1995). Improving the readability of time-frequency and time-scale representations by the reassignment method. IEEE Trans. Signal Process..

[20] Daubechies I., Lu J., Wu H.-T. (2011). Synchrosqueezed wavelet transforms: An empirical mode decomposition-like tool. Appl. Comput. Harmon. Anal..

[21] Oberlin T., Meignen S., Perrier V. The Fourier-based synchrosqueezing transform. Proceedings of the 2014 IEEE International Conference on Acoustics, Speech and Signal Processing (ICASSP).

[22] Huang Z.-L., Zhang J., Zhao T.-H., Sun Y. (2015). Synchrosqueezing S-transform and its application in seismic spectral decomposition. IEEE Trans. Geosci. Remote Sens..

[23] Oberlin T., Meignen S., Perrier V. (2015). Second-order synchrosqueezing transform or invertible reassignment? Towards ideal time-frequency representations. IEEE Trans. Signal Process..

[24] Pham D.-H., Meignen S. (2017). High-order synchrosqueezing transform for multicomponent signals analysis—With an application to gravitational-wave signal. IEEE Trans. Signal Process..

[25] Wang S., Chen X., Cai G., Chen B., Li X., He Z. (2013). Matching demodulation transform and synchrosqueezing in time-frequency analysis. IEEE Trans. Signal Process..

[26] Yu G., Yu M., Xu C. (2017). Synchroextracting transform. IEEE Trans. Ind. Electron..

[27] Yu G. A geometry study on reassignment method and synchrosqueezing transform. Proceedings of the 2018 Chinese Automation Congress (CAC).

[28] Fourer D., Auger F., Hu J. (2015). Reassigning and synchrosqueezing the Stockwell transform: Complementary proofs.

[29] Liu W., Liu Y., Li S., Zhai Z. (2023). Demodulated synchrosqueezing S-transform and its application to machine-fault diagnosis. Meas. Sci. Technol..

[30] Feng Z., Qin S., Liang M. (2016). Time–frequency analysis based on Vold-Kalman filter and higher order energy separation for fault diagnosis of wind turbine planetary gearbox under nonstationary conditions. Renew. Energy.

[31] Feng Z., Chen X., Liang M., Ma F. (2015). Time–frequency demodulation analysis based on iterative generalized demodulation for fault diagnosis of planetary gearbox under nonstationary conditions. Mech. Syst. Signal Process..

[32] Liu D., Cui L., Cheng W. (2023). A review on deep learning in planetary gearbox health state recognition: Methods, applications, and dataset publication. Meas. Sci. Technol..

[33] Chen S., Liu Z., He X., Zou D., Zhou D. (2024). Multi-mode fault diagnosis datasets of gearbox under variable working conditions. Data Brief.

